# An In Vitro Dosimetry Tool for the Numerical Transport Modeling of Engineered Nanomaterials Powered by the Enalos RiskGONE Cloud Platform

**DOI:** 10.3390/nano12223935

**Published:** 2022-11-08

**Authors:** Nikolaos Cheimarios, Barbara Pem, Andreas Tsoumanis, Krunoslav Ilić, Ivana Vinković Vrček, Georgia Melagraki, Dimitrios Bitounis, Panagiotis Isigonis, Maria Dusinska, Iseult Lynch, Philip Demokritou, Antreas Afantitis

**Affiliations:** 1 NovaMechanics Ltd., Nicosia 1070, Cyprus; 2 Institute for Medical Research and Occupational Health, 10 000 Zagreb, Croatia; 3 Hellenic Military Academy, 16673 Vari, Greece; 4 Center for Nanotechnology and Nanotoxicology, HSPH-NIEHS Nanosafety Research Center, Department of Environmental Health, Harvard School of Public Health, Boston, MA 02115, USA; 5 Department of Environmental Sciences, Informatics and Statistics, Ca’ Foscari University of Venice, 30172 Venice, Italy; 6 Department of Environmental Chemistry, Health Effects Laboratory, NILU—Norwegian Institute for Air Research, 2007 Kjeller, Norway; 7 School of Geography, Earth and Environmental Sciences, University of Birmingham, Birmingham B15 2TT, UK

**Keywords:** in vitro dosimetry, distorted grid model, nanotoxicity, Au and Ag nanoparticles, Enalos Cloud Platform

## Abstract

A freely available “in vitro dosimetry” web application is presented enabling users to predict the concentration of nanomaterials reaching the cell surface, and therefore available for attachment and internalization, from initial dispersion concentrations. The web application is based on the distorted grid (DG) model for the dispersion of engineered nanoparticles (NPs) in culture medium used for in vitro cellular experiments, in accordance with previously published protocols for cellular dosimetry determination. A series of in vitro experiments for six different NPs, with Ag and Au cores, are performed to demonstrate the convenience of the web application for calculation of exposure concentrations of NPs. Our results show that the exposure concentrations at the cell surface can be more than 30 times higher compared to the nominal or dispersed concentrations, depending on the NPs’ properties and their behavior in the cell culture medium. Therefore, the importance of calculating the exposure concentration at the bottom of the cell culture wells used for in vitro arrays, i.e., the particle concentration at the cell surface, is clearly presented, and the tool introduced here allows users easy access to such calculations. Widespread application of this web tool will increase the reliability of subsequent toxicity data, allowing improved correlation of the real exposure concentration with the observed toxicity, enabling the hazard potentials of different NPs to be compared on a more robust basis.

## 1. Introduction

As hazard characterization and risk assessment are an integral part of the lab-to-market innovation path of nano-enabled products, assessment of their safety should follow a multimethod tiered approach from the early phases of research to the final product; from the characterization of physicochemical and biological properties of nanoparticles (NPs) (Tier 1) via the evaluation of their interaction with biological systems using decisive in vitro/in vivo dosimetry (Tier 2) to, finally, the identification of their mode of action (Tier 3). Despite excellent progress towards robust methods for determination of the NPs’ properties for toxicity studies [1,2], NPs’ interactions with and uptake by cells [3,4], and the resulting hazard effects [5,6,7] necessary for reliable risk assessment, there are still huge variations in how in vitro studies are performed, how data are reported, and especially how and whether dose–response relationships can be determined given the dynamic nature of particle dispersions [8] and the enormous variability within and between cells in terms of NPs uptake [3,9]. Uncertainty around the actual internalized cellular concentration limits the reuse of existing data for read-across analyses. A significant obstacle for characterization is the requirement to prepare stable dispersions not only in the optimal solvent, but also in the biological medium used during evaluation at Tier 2 and Tier 3 levels, typically a high-salt- and high-protein-containing medium leading to the potential for particle agglomeration and biomolecule corona formation [10,11]. In particular, the physicochemical properties of NPs affect their colloidal stability and behavior during standard in vitro/in vivo nanotoxicity testing, particularly influencing the number of particles delivered to cells, tissues and organs [12,13,14,15]. It has recently been shown [16] that implementation of a freeze–thaw protocol altered the presence of agglomerates in the particle size distribution of fresh dispersions up to 35% and modulated the dosimetry of the particles, and this effect was especially problematic in medium without serum.

As part of the Tier 2 testing phase, decisive “in vitro dosimetry” represents one of the most critical challenges for hazard and risk assessment, and it is directly dependent on the stability, transformation, uptake, transport and biological effects of the NPs under investigation. Recently, an integrated “in vitro dosimetry” approach has been proposed, which comprises of dispersion preparation, dispersion characterization and numerical fate and transport modeling of NPs to derive the particle concentration delivered at the site of their action [17], building on significant earlier efforts to establish particokinetics for in vitro toxicity assessment [18,19] of NPs of different sizes, compositions and shapes.

Nanotoxicity testing using in vitro assays requires the NPs to be dispersed in cell culture medium and applied to multiwell cell culture plates for assessment of impacts on cell viability or expression of various compounds, such as reactive oxygen species, cytokines or other markers of response to the present of the NPs, over time. There are numerous techniques and protocols for dispersing NPs in aqueous media that should be harmonized [20]. Various endpoints are measured during in vitro testing following cell exposure to NPs, commonly over 24–72 h, while the dose–response (or more correctly for in vitro studies, the NPs concentration–response) analysis describes the cause–effect relationship [17]. However, the effective (or actual exposure) concentration is not necessarily equal to the available (nominal) dose in the case of NPs, since the cells seeded in the plate well will only interact with the NPs near the bottom of the plate, i.e., those NPs that can attach to the cell surface. Therefore, for correct reporting of the NPs’ dosage regimen and proper explanation and interpretation of results gathered during in vitro testing, the effective (actual concentration at the cell surface) particle concentration should be determined [21,22]. The approaches and methods for determination of NPs interactions with cells, and the consequent hazard effects, have been established and refined over the last decade, with a central focus on characterization of the dispersion stability, the attachment to cells and the toxicity arising from cellular accumulation. The two primary transport mechanisms of NPs to the cell surface during in vitro assays are diffusion and sedimentation. Both mechanisms are significantly affected by the NPs’ size and the effective density (including both primary particles and agglomerates). If these properties are known, the surface-available exposure concentrations can be calculated via mathematical models [22,23]. The most popular models that have been developed to date are the in vitro sedimentation, diffusion and dosimetry (ISDD) model [22,24,25,26] and the distorted grid (DG) model [17,27,28]. Both are based on differential equations and require information on the NPs’ size and effective density as input data. Other available models are based on stochastic approaches, such as the direct simulation Monte Carlo (DSMC) model [29] or the Agglomeration–Diffusion–Sedimentation–Reaction Model (ADS-RM) [30]. Compared to the ISDD models, the DG model is more generalized, offering modeling of particles that undergo dissolution over time, as well as a variable “stickiness” boundary conditions at the bottom through a Langmuir isotherm process. Compared to the DSCM models, DG needs a minimum amount of input information compared to the various factors and empirical mechanisms needed by the DSCM models [31], which limits their use.

However, none of the models able to compute the effective dose at the bottom of the cell culture plate wells and available to the cell surface are implemented as a software, licensed or open source, and thus these models are not available to the research community. Thus, widespread implementation of these models is hampered and much of the literature continues to base dose–response relationships and interpretation of toxicity data on the dispersion concentration only, which is a great drawback, since the dispersion concentration can be very different compared to the particle concentration at the cell surface. Our inspection of the Web of Science and PubMed databases, performed in July 2022, and searching for nanotoxicity studies revealed that most of the nanotoxicity studies did not consider employing mathematical models to calculate the effective NPs concentrations. In the case of the DG model, we found less than 130 scientific papers published in the period from 2017 to 2022 that applied this model to determine actual cellular exposure concentrations of tested NPs. None of these 130 studies developed user-friendly software but rather used MatLab or similar programming language to calculate the NPs’ effective dose. Moreover, a literature search in the Web of Science and PubMed databases using keywords “nano*” AND “toxic*” AND “in vitro” resulted in 26,827 published papers, while a refined search including keywords related to any of the mathematical models described above (i.e., DG, ISDD, DSCM or ADS-RM) derived only 63 papers. Among these publications, there was no consensus on the most relevant dose metrics for engineered nanomaterials, and different outputs were reported, including NP fraction deposited at the bottom of the well, NP mass per surface area of wells and effective delivered dose, etc. That said, less than 0.5% of all in vitro nanotoxicity studies provided any data related to the calculation of the effective NPs concentration at the cell surface (for adherent cells), but instead reported nominal NPs concentrations (dispersed concentration), assuming that the particles remained homogenously dispersed throughout the volume of the medium. Such assumptions may lead to false interpretation of the data obtained from nanotoxicity studies, thus significantly hampering risk assessment of nano-enabled products.

To address this gap, we aimed to develop a free web-based application to be used for calculation of the effective NPs dose in in vitro assays. The web application, termed as an “in vitro dosimetry” web application, is based on the DG model and allows calculation of the mass-, number- and surface area-based NPs concentrations in the cellular microenvironment throughout the duration of cellular exposure to different NPs. The results are in good agreement with experimental data, as has been shown previously [31,32]. By performing a series of in vitro experiments for six different NPs, with Ag and Au cores, we demonstrate the utility of the web application and confirm the disparity between the dispersion concentration and the particle concentration in the vicinity of the cells. In some cases, the exposure concentration at the cell surface was found to be more than 30 times higher than the nominal concentration in the dispersion. This large difference greatly affects the final conclusions about toxicity and hazards of the particular NPs, and indeed, the hazard potency per NP, as if more particles are actually in contact with the cells than expected from the dispersion concentration, then the actual toxicity per particle is lower than would have been assumed based on the dispersion concentration.

## 2. RiskGONE Instance of the Enalos Cloud Platform

The web application was developed as part of the Horizon 2020 project RiskGONE (https://riskgone.eu/, accessed on 31 October 2022), instance of Enalos Cloud Platform, a suite of user-friendly web-based tools, developed within various EU-funded projects for assessment of the risks associated with engineered nanomaterials and deployed via the cloud: http://www.enaloscloud.novamechanics.com/index.html (accessed on 31 October 2022). One of the major goals of the RiskGONE project is to implement a nanoinformatics-driven decision-support strategy that promotes nanosafety based on innovative in silico methods, models and tools, which will reduce reliance on animal testing following the Three Rs principle (reduction, replacement and refinement of animal testing). Presenting and deploying the developed nanoinformatics tools as freely available, user-friendly web applications, accompanied by appropriate model documentation and user guidance, significantly increases model accessibility and usage, even by non-experts. Understanding and utilizing these nanoinformatics tools can bridge the gap between nanosafety-related researchers, regulators and industry, thus accelerating the industrial and commercial applications of NPs while minimizing their hazardous impact on environmental and human health.

**Figure 1:**
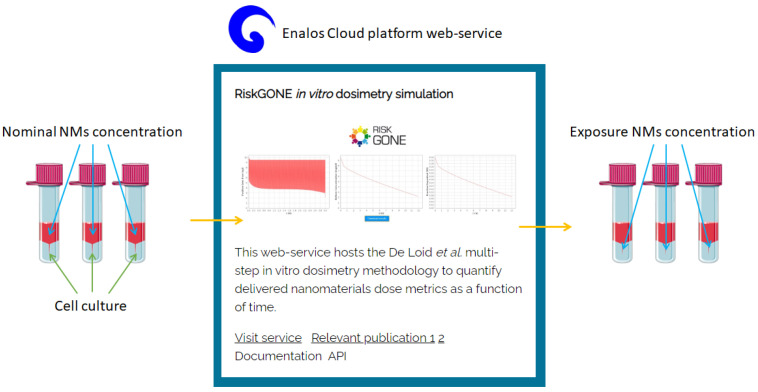
Τhe RiskGONE instance of the Enalos Cloud Platform, showing the RiskGONE “in vitro dosimetry” simulation web application. Clicking on the application opens the model webpage, which consists of a description of the model and step-by-step instructions regarding the inputs needed: http://enaloscloud.novamechanics.com/riskgone/InVitroDosimetry/ (accessed on 31 October 2022).

The “in vitro dosimetry” tool was developed as a single web-page application using Java and the zk framework. All underpinning calculations were performed on NovaMechanics servers, and the results generated are presented as tabular and graphical outputs for easy download. The application is available as part of the RiskGONE instance of the Enalos Cloud Platform, as shown in Figure 1: http://www.enaloscloud.novamechanics.com/riskgone.html (accessed on 31 October 2022). The “in vitro dosimetry” application is detailed in the following paragraphs.

### 2.1. Required Data/Input to the “In Vitro Dosimetry” Simulation

**Figure 2:**
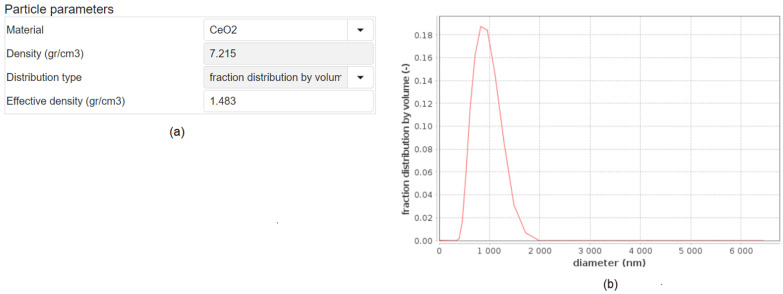
The Particle parameters section of the “in vitro dosimetry” web application. (**a**) The basic input parameters. The user can select from various NPs whose parameters are pre-loaded, or can define new NPs and input the required information on density and effective density themselves, as well as the particle size distribution type (i.e., volume distribution (mass) or particle number distribution). (**b**) Illustration of the particle size distribution of the CeO_2_ NPs dispersed in Eagle’s minimum essential medium (EMEM) cell culture medium with 10% fetal bovine serum presented as the fraction distribution by volume for the specific NP.

The input for the web application consists of three sections: the Particle parameters, the Solvent parameters and the Simulation parameters, which are presented sequentially. Auxiliary to these is the optional Advanced parameters section. In the Particle parameters section, the user defines the input regarding the particular NPs under study (see Figure 2). The available NPs are CeO_2_, SiO_2_, Fe_2_O_3_, TiO_2_, CuO, ZnO, Au, Ag, FePO_4_ (anhydrous) and “User defined …”. If selecting “User defined …”, the user must provide information on the NP’s density (Figure 2a). Otherwise, this part is automatically filled with the density of the available NPs when selected. The user must also provide the “effective density” of the NPs with the solvent. DeLoid et al. have developed an easy-to-use method to measure the effective density of NPs in biological media [32]. In addition, the fraction distribution by volume (i.e., based on the relative mass of the particles) or the % number-weighted size (i.e., the number of particles in each size bin) versus the diameter (nm) for the specific NP is needed (Figure 2b). Note that further information on the difference between volume- and number-based NPs size distributions, and methods for how these can be determined, are provided in [33], and the impact of which approach is used on the reported toxicity is illustrated in [33,34]. Figure 2a shows the basic input parameters for CeO_2_ NPs along with the (size) fraction distribution by volume in Figure 2b.

**Figure 3:**
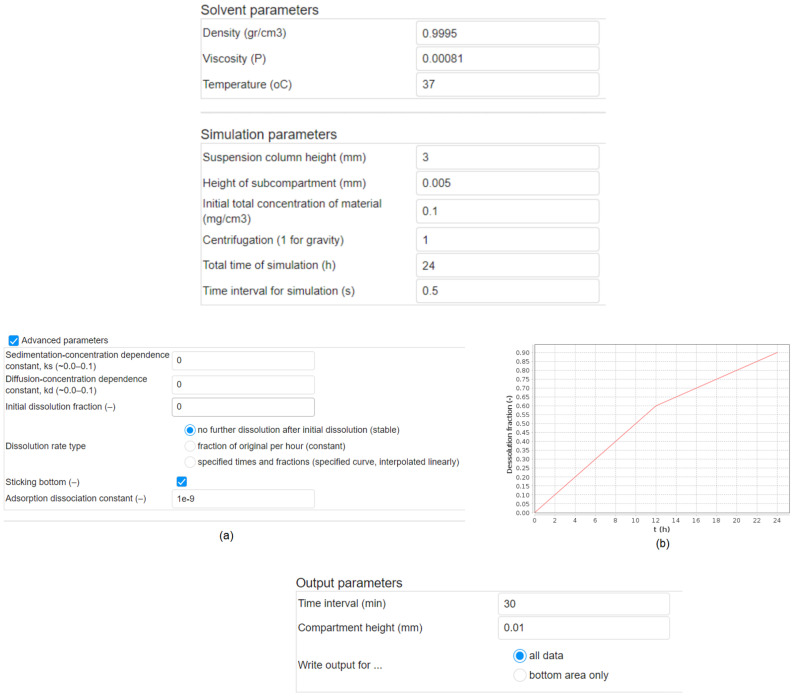
The Solvent parameters needed as input, shown in this case for ultrapure water, and the Simulation parameters section, which are used to describe the vessel in which the NPs are dispersed or the cell culture dish in which the NPs are administered. The Advanced parameters section. (**a**) The basic input which gives information on the rates of sedimentation and diffusion (default values are set to zero) and describes the dissolution status of the NPs’ dispersion and the rate of dissolution (if any), and whether an attachment factor is required, for example, due to the presence of an adherent layer of cells at the bottom of the well. (**b**) Example of Dissolution fraction versus time, t (in hours), if the user selects to specify times and fractions in Dissolution rate type. The Output parameters section, where the user selects the time period for the simulation, the height of the exposure vessel (e.g., the cell culture dish) and whether they want to record the NPs’ concentrations throughout the vessel or only for the bottom layer.

For the Solvent parameters, the user defines solvent-related information, in particular density (gr/cm^3^), viscosity (P) and temperature (°C), while for the Simulation parameters, simulation-related information and in particular the height (mm) of the suspension well or column (the “Height” in Figure S1 of the Supplementary Material), the height, h (mm) of the specific compartment (see Figure S1 of the Supplementary Material), the initial concentration (mg/cm^3^) of the NPs, the total simulation time (hours) and the time interval (Δt − sec) of the simulation. All of this information is shown in Figure 3, for the CeO_2_ NPs dispersed in ultrapure water.

Finally, the Advanced parameters section (see Figure 3) allows the user to define specific input properties concerning the simulation. The latter includes the sedimentation and the diffusion coefficients. The sedimentation coefficient takes into account the dependence of the sedimentation process on the initial particle concentration, as this varies in a nonlinear way [35] as presented in Equation (1).

(1) $$S_{i,j}^{\prime }=\frac{S_{i,j}}{1+k_{s}C_{i,j}}$$

Likewise, the nonlinear concentration dependence factor for the diffusion coefficient is given by:

(2) $$D_{i,j}^{\prime }=\frac{D_{i,j}}{1+k_{d}D_{i,j}}$$

where *k_s_* is the sedimentation-concentration dependence constant and *k_d_* the diffusion-concentration dependence constant. Typical values for both *k_s_* (-) and *k_d_* (-) are 0 to 0.1.

One of the challenges of determining NPs dosimetry is the dynamic nature of NPs in dispersion. For example, while the majority of metal and metal oxide NPs are insoluble or poorly soluble in aqueous solution, for those NPs that may undergo partial dissolution on the timescale of the cellular exposure experiments (24–72 h) such as Ag, Zn and Cu NPs, for example, dissolution must be considered in the model. The rates of dissolution over time can be estimated theoretically in specific cases [30] or via empirical measurements of NPs dissolution by techniques such as ICP-MS [36,37]. Solubilization may affect the particle size and size distribution. The user must thus define the initial dissolved fraction, which has zero as the default value. For the dissolution rate, three selections are available: (i) no further dissolution after the initial dissolution, (ii) fraction of original dissolution per hour where a constant value is inserted for the rate and specified times, or (iii) fractions as per the ones shown in Figure 3b. Furthermore, the DG model offers a variable “stickiness” boundary condition at the bottom of the column, which is used to represent the attachment to adherent cells at the bottom of the well, which is implemented using a Langmuir isotherm adsorption model, as described in detail in [17]. The adsorption dissociation constant is used to compute the surface coverage in the Langmuir isotherm adsorption model, which is calculated using:

(3) $$\theta =\frac{\left[P\right]}{K_{D}+\left[P\right]}$$

where *θ* is the coverage, *K_D_* is the adsorption dissociation constant and [*P*] (mol cm^−3^) is the particle molar concentration in the bottom compartment, which is computed as:

(4) $$\left[P\right]=\frac{C_{p}\;}{N_{A}\rho_{EV}\left(\frac{4}{3}\pi r^{3}\right)}$$

where *C_p_* is the mass concentration of NPs in the bottom compartment, *N_A_* is Avogadro’s number and *r* is the particle radius (NP or agglomerate).

### 2.2. Output

The Output section is shown in Figure 3, where the user can define the time intervals during the simulation, the concentrations in each compartment, as well as selecting which dose metrics, i.e., derived mass, particle number and surface area dose, to save during the course of the simulation. There is also the possibility to save the data for the whole suspension column height or just for the bottom layer in contact with the cells.

## 3. Case Studies

Six different NPs are used as case studies for the demonstration and proof-of-concept of the “in vitro dosimetry” web application. All NPs were designed, prepared and characterized in the laboratories of the Institute for Medical Research and Occupational Health, Zagreb, Croatia. The set includes four different silver NPs (AgNP), coated with cysteine (CYS), glutathione (GSH), bis(2-ethylhexyl) sulfosuccinate (AOT) and poly-*L*-lysine (PLL) and two different gold NPs (AuNP), coated with CYS and GSH, to demonstrate the use of the “in vitro dosimetry” web application for NPs of the same size but with different surface coatings and surface charges. Preparation, characterisation and nanotoxicity evaluation of CYS- and GSH-coated AgNP and AuNP have been described recently, along with their toxicity to the murine fibroblast cell line (L929, ATCC^®^ CCL- 1TM) [38]. Toxicity of the AOT- and PLL- coated AgNP was also performed using human keratinocytes (HaCaT cell line). Six nominal concentrations were considered for the GSH- and CYS-coated AuNP and AgNP, namely 0.001, 0.005, 0.01, 0.05, 0.1 and 0.3 mg cm^−3^, and eight concentrations were utilized for the AOT- and PLL-AgNP, namely 0.00125, 0.0025, 0.005, 0.01, 0.02, 0.04, 0.08 and 0.16 mg cm^−3^. For the purpose of this study, the AOT- and PLL-coated AgNP were freshly prepared according to the procedure described elsewhere [39] and characterized in the medium used for cell experiments (Eagle’s minimum essential medium (EMEM) supplemented with 10% fetal bovine serum) by means of hydrodynamic diameters (*d_H_*) using dynamic light scattering (DLS) and zeta potential (in mV) by electrophoretic light scattering (ELS) experiments. Physicochemical characteristics of the tested NPs are given in Table 1.

The NPs’ toxicity to human keratinocytes (HaCaT cell line) was evaluated by means of the MTT cell viability assay and oxidative stress response using 2′,7′-dichlorofluorescein diacetate (DCFH-DA) staining. In the case of CYS- and GSH-coated AgNP and AuNP, data obtained during a previous study [24] were used, i.e., *d_H_* and zeta potential values from DLS and ELS measurements, respectively, as well as cell viability of NPs-treated L929 cells obtained using the MTT assay. Moreover, all selected NPs were tested for dissolution in the dispersion medium, i.e., the EMEM culture medium used for cell experiments according to the protocol described elsewhere [38]. Physicochemical characteristics of all tested NPs are given in Table S1 of the Supporting Material. All experimental details related to the synthesis and characterization of the AOT- and PLL-coated AgNP are given in the Supporting Material, while materials and synthesis protocols used for CYS- and GSH-coated AgNP and AuNP can be found in reference [38]. For all in vitro experiments, the differences between treatments for the cell viability results were tested using simple and repeated measures ANOVA, followed by a Fisher LSD post hoc test when significant differences were found (*p* < 0.05). The homogeneity of variance was tested using the Levene test. The level of significance (*p* < 0.05) is indicated by the asterisks (*) for differences between NP treatments and controls (non-treated cells).

To run the in vitro dosimetry simulation, the input Particle parameters were “User defined”, according to the values given in Table 1, and the NPs’ size fraction distribution by volume was obtained by DLS measurements, as shown in Table S1 and Figure S1 (Supporting Material). The input parameters for Solvent (cell culture medium) were set at 0.9995 g cm^−3^, 0.00081 P and 37 °C for the effective density, viscosity and temperature, respectively. The Simulation parameters were dependent on the design of the cell culture plates used for cell experiments. The MTT experiments were conducted in 96-well plates with 100 μL as the total volume of liquid per well, and the suspension column height was 3 mm. The height of the sub-compartment was set as 0.005 mm, and the total simulation time was 24 h in all experiments, equal to the duration of cell treatment with the different NPs. In the case of AOT- and PLL-AgNP, the default value for the time interval was used i.e., 0.5 s, while for CYS- and GSH-coated NPs, this value was increased to 2 s to reduce the time required for the simulation to complete. As the dissolution of all tested NPs was experimentally shown to be negligible, dissolution was not included in the simulation. For the Output parameters, a time interval of 60 min and compartment height of 0.01 mm were set. All data input used are given in Table 1 as well as in the Supporting Material (see Table S1 and Figure S2).

## 4. Results and Discussion

**Figure 4:**
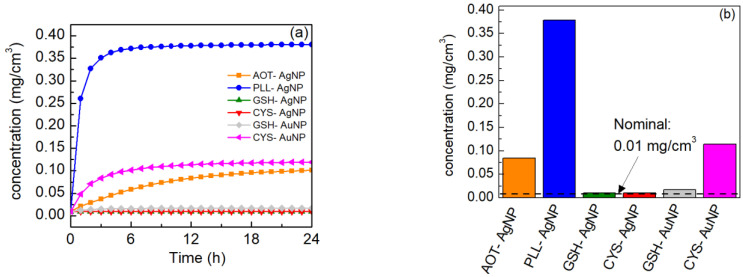
Calculated values of different NPs with the same nominal concentration (0.01 mg/cm^3^) deposited per surface area of a 96-well plate. (**a**) Evolution of sedimentation of different NPs at the bottom of the wells of the cell culture plates expressed as mass concentration, and (**b**) the median value of the amount of NPs that reached the bottom compartment of the assay plates for 1 h time interval expressed as mass concentration.

Evolution of the NPs’ concentration at the bottom of the wells of the cell culture plates used for the in vitro assays, as simulated by the “in vitro dosimetry” web application, can be seen in Figure 4a. After obtaining the NPs concentrations in the bottom compartment of the assay plates for a 1 h time interval, the median value was calculated. The latter is shown in Figure 4b. Our calculation demonstrated that accumulation of NPs at the bottom of the wells can be considered a complex process and cannot be explained only by the NPs’ size, but it is also influenced by the surface charge and the type of surface coating. For example, sedimentation of the PLL-AgNP reached equilibrium in less than 8 h, resulting in the highest number of NPs at the bottom of the wells compared to all other NPs tested. A similar sedimentation rate was observed for GSH- and CYS-AgNPs, while the AOT-AgNP sedimentation was much slower and did not reach a plateau until almost 24 h. Despite the deposition of larger NPs being expected to be enhanced by the impact of gravity compared to smaller NPs, such a trend was not obvious as a smaller amount of the larger-sized AOT-AgNP (48.1 ± 2.0 nm) settled to the well bottom compared to the smaller CYS-AuNP (18.7 ± 11.1 nm). Comparison of the nominal (dispersion concentration) and the effective concentrations in the bottom layer close to the cells, as calculated by the “in vitro dosimetry” web application, indicates that the cells are exposed to a much higher concentration of some of the NPs than what can be assumed from the reported nominal values, while this exposure amount is dependent on the NPs’ behavior in the dispersion, i.e., sedimentation rate and possible agglomeration, which is unique for each NP type (see Figure 4b).

All data computed for the selected NPs are gathered into Tables S2 and S3 of the Supporting Material. It is clear that the cellular exposure varies significantly between different NP types for the same nominal mass concentration in the initial dispersion. In the case of PLL-AgNP, the effective concentration was almost 40 times higher than the nominal one (the initial dispersion concentration), while AOT-AgNP and CYS-AuNP showed similar effective concentrations despite having different sizes and surface coatings. In other cases, such as GSH-AgNP, CYS-AgNP and CSH-AuNP, the effective concentration almost coincides with the nominal one, confirming that the web application can also be used to confirm where literature data do not require concentration correction before re-use for modeling and other purposes. The different behaviors in terms of nominal and cellular exposure concentrations of the NPs is attributed to the variations in sizes and compositions (see Table 1 for comparison) of the NPs: for example, when the calculated in vitro exposure concentration in the bottom layer of the wells is converted into the particle number per unit area concentrations, the number of deposited AOT-AgNP is much smaller than the number of PLL-AgNP for the same nominal mass concentration (i.e., mg Ag/cm^3^) used for cell treatment, despite the fact that their core particle sizes do not differ very much (see Table 2, *d_H_* of 48.1 ± 2.0 nm for AOT-AgNP and 24.2 ± 2.6 nm for PLL-AgNP). One potential explanation for this difference is the molecular weight differences between the coatings and their relative proportions of the overall particle mass: AOT has a molecular weight of 438.6 g/mol while that of PLL is much larger, depending on the polymer size (e.g., 4700 g/mol for a degree of polymerization of 30). Additionally, the better colloidal stability and electrostatic repulsion between negatively charged AOT-AgNP in the cell culture medium would also lead to slower sedimentation compared to PLL-AgNP, which may be electrostatically destabilized in the cell culture medium by interaction with negatively charged species (e.g., proteins).

**Figure 5:**
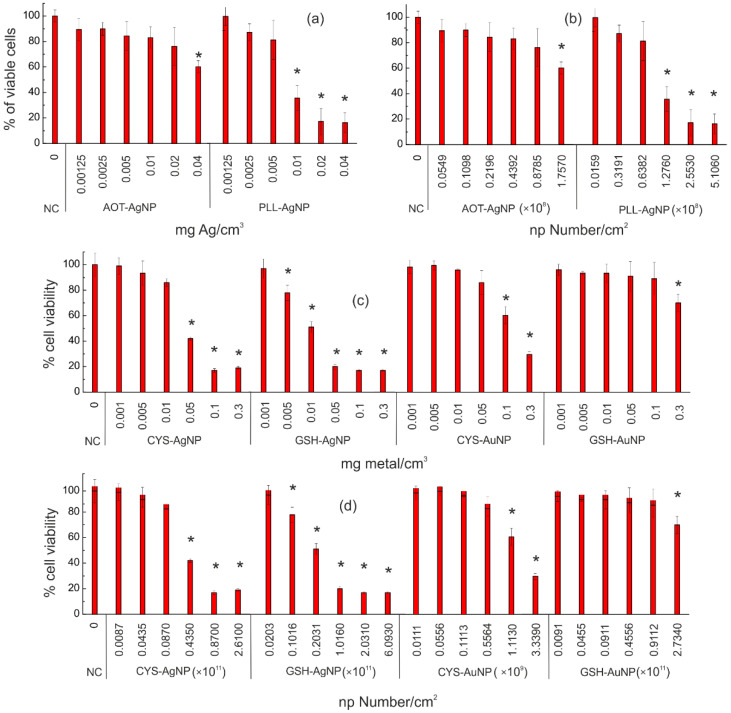
L929 cell viability after 24 h treatment with different concentrations of AOT- and PLL-coated AgNP (**a**,**b**), and L929 cell viability after 24 h treatment with different concentrations of CYS- and GSH-coated AgNP and AuNP (**c**,**d**). In (**a**,**c**), concentrations are expressed as the nominal concentrations, i.e., mg of metal per cm^3^ of cell culture medium in the initial dispersion, while in (**b**) and (**d**) the results are presented as the response in cell viability using the calculated number of NPs deposited per unit area of the well bottom determined utilizing the “in vitro dosimetry” web application. Percentages of viable cells are expressed relative to the negative control (NC, cells treated with medium only). The values marked with an asterisk (*) differ significantly from the negative control (*p* < 0.05).

Figure 5 presents the results of the cytotoxicity evaluation by comparing NP concentrations expressed as the nominal concentration (initial dispersion concentration in mass/volume) and the calculated number of deposited NPs per unit area of well plate, determined using the “in vitro dosimetry” web application. These figures perfectly display the pitfalls of using nominal NPs concentrations to track and compare NPs toxicities. Figure 5a presents the viability of L929 cells in response to different nominal concentrations of AOT-AgNP and PLL-AgNP, while the calculated NP number per unit area of the well bottom is used in Figure 5b. When using nominal NPs concentrations, a relatively high toxicity of PLL-AgNP can be observed. This was also confirmed by the “in vitro dosimetry” web application calculations, as a lower number of PLL-AgNP deposited on the cell surface (1.28 × 10^8^ particles) decreased cell viability by ca. 60%, compared to 1.76 × 10^8^ particles of AOT-AgNP deposited on the cell surface, which decreased the cell viability by less than 40%. Comparison of results obtained for the treatment of L929 cells with CYS- and GSH- coated AgNPs and AuNPs is extremely interesting and indicates the importance of the “in vitro dosimetry” calculation. Given only nominal concentrations of CYS-AgNPs and GSH-AgNPs (Figure 5c), CYS-AgNP might appear to be more toxic than CYS-AuNPs, but the number of viable cells was significantly reduced already at 1.11 × 10^9^ particles of CYS-AuNP per cm^2^ of cell surface in comparison with the much higher number of CYS-AgNP needed for significant cell viability reduction (4.35 × 10^10^ NPs per cm^2^). Therefore, the initial conclusion obtained when using nominal NPs concentrations completely changed after “in vitro dosimetry” calculations in some cases, depending on the potential for increased concentration of the NPs at the cell surface during the exposure. However, this was not the case for GSH-coated NPs, as the same conclusion about the toxicity of GSH-AgNPs and GSH-AuNPs to L929 cells can be reached when concentrations are expressed either as the nominal NPs concentration (Figure 5c) or as the NPs’ number per cell surface area (Figure 5d). This highlights the need to consider in vitro dosimetry metrology on a case-by-case basis and indeed to compare the different dose-metrics to fully interrogate the resulting toxicity data.

The toxicity results are affected by the stability and behavior of the NPs in the exposure medium, among other factors. Therefore, the input parameters for our web application version of the in vitro dosimetry model, which is based on the DG model, should be obtained for the test NPs dispersed in the exposure medium used for in vitro experiments, since serum proteins and other dispersion parameters can affect the dose of NPs reaching the cells [16]. Finally, it is important to note that NPs’ cytotoxicity is a complex process and can occur by several mechanisms and does not depend only on the number of deposited NPs, but also on factors such as cellular attachment and uptake, which are not covered by the current model. While the limitations of our web application must be acknowledged, the use of dosimetry calculations to properly assess NPs toxicity and hazard is a huge step forward in the efforts to standardize protocols and ensure result comparability and reliability. Future work will include the integration of the “in vitro dosimetry” web application with a model we developed previously for the prediction of NPs’ cellular association (as the first step of NPs uptake into cells) [41] and for toxicity prediction, as part of an in silico integrated approach to testing and assessment (IATA) for hazard and risk assessment of engineered nanomaterials.

## 5. Conclusions

Here, we presented a web application, based on the distorted grid (DG) model, for the calculation of the effective concentration of NPs that can come into contact with an adherent cellular layer at the bottom of the cell culture well during in vitro assays. The true importance of the “in vitro dosimetry” application can be best described by highlighting the difference between reporting experimental results based on nominal (initial dispersion concentration) and effective (actual number of particles in contact with the cells at the bottom of the well) NPs’ concentration metrics. In standard nanotoxicity experiments, the difference in effect between different NPs are based on the nominal concentrations expressed as mass per volume of cell culture medium above the cells. However, the “in vitro dosimetry” calculator enables users to inspect whether the same nominal concentrations of different NPs will have a similar or different mass, particle number or particle surface area of NPs that actually reach the cell surface as a result of the processes occurring in the solution during the exposure, including NPs agglomeration, sedimentation and diffusion. It is essential to present the effective concentration of particles reaching the cell surface as accurately as possible, since only those NPs that reach the bottom of the cell culture well during the exposure duration, i.e., reach the cell surface, will display biological and toxicological effects in cells.

From an application point of view, our analysis, with the effective concentrations from the in vitro dosimetry experiments, shows that AuNP are not less toxic than AgNP, which is in contrast to the results acquired when the nominal concentrations are used. As the deposition of different NPs during in vitro experiments is not straightforward and cannot be predicted just by considering the hydrodynamic size or surface charge of the NPs, calculation of the effective NPs concentration reaching the cells through use of a DG model is recommended for each NP of interest. Our web application—available at http://enaloscloud.novamechanics.com/riskgone/InVitroDosimetry/ (accessed on 31 October 2022)—significantly reduces the effort and time required for such calculations and can be applied retrospectively to cytotoxicity data based on the description of the NPs and the cell culture conditions utilized. Future steps, already underway, include the integration of the “in vitro dosimetry” web application with the NanoPharos database, https://db.nanopharos.eu/Queries/Datasets.zul (accessed on 31 October 2022), as well as with the Enalos Cloud Platform, http://www.enaloscloud.novamechanics.com/ (accessed on 31 October 2022), to extend the domain of applicability of existing tools and applications.

## Figures and Tables

**Table 1:** Properties of the tested NPs used as input for the “in vitro dosimetry” web application.

Nanoparticle	Material	Density (g/cm^3^)	Effective Density * (g/cm^3^)	*d_H_* (nm) (% Volume)	ζ Potential (mV)
AOT-AgNP	Ag	10.49	8.58	48.1 ± 2.0 (100%)	−35.1 ± 0.7
PLL-AgNP	Ag	10.49	8.58	24.2 ± 2.6 (100%)	47.6 ± 2.4
CYS-AgNP	Ag	10.49	8.58	6.6 ± 1.5 (100%)	−44.5 ± 6.2
GSH-AgNP	Ag	10.49	8.58	4.5 ± 1.7 (100%)	−19.5 ± 6.1
CYS-AuNP	Au	19.30	17.73	18.7 ± 11.1 (100%)	−32.3 ± 4.5
GSH-AuNP	Au	19.30	17.73	3.9 ± 1.2 (100%)	−41.2 ± 6.4

* Obtained from literature. Ag reference: Tadjiki et al. 2017 [40], Au reference: DeLoid et al. 2017 [17].

**Table 2:** Comparison of median values of calculated dosimetric parameters for the tested AgNP and AuNP at the nominal concentrations of 0.005 and 0.01 mg cm^−3^ used in the toxicity experiments. All values correspond to the effective NPs concentrations calculated at the bottom compartment of the cell culture plates (cellular microenvironment).

Calculated Value	Nominal conc. (mg/cm^3^)	AOT-AgNP	PLL-AgNP	GSH-AuNP	GSH-AgNP	CYS AuNP	CYS AgNP
Mass concentration of NPs at well bottom (mg/cm^3^)	0.005	0.042	0.189	0.008	0.005	0.057	0.005
	0.01	0.084	0.378	0.017	0.010	0.114	0.010
Mass per unit area of well (mg/cm^2^)	0.005	4.192 × 10^−5^	1.891 × 10^−4^	8.353 × 10^−6^	5.019 × 10^−6^	5.676 × 10^−5^	5.034 × 10^−6^
	0.01	8.384 × 10^−5^	3.782 × 10^−4^	1.671 × 10^−5^	1.004 × 10^−5^	1.135 × 10^−4^	1.007 × 10^−5^
NPs number at well bottom (cm^−3^)	0.005	2.196 × 10^10^	6.382 × 10^11^	4.556 × 10^12^	1.016 × 10^13^	5.564 × 10^10^	4.350 × 10^12^
	0.01	4.392 × 10^10^	1.276 × 10^11^	9.112 × 10^12^	2.031 × 10^13^	1.113 × 10^11^	8.701 × 10^12^
NPs number per unit area of well (cm^−2^)	0.005	2.196 × 10^7^	6.382 × 10^7^	4.556 × 10^9^	1.016 × 10^10^	5.564 × 10^7^	4.350 × 10^9^
	0.01	4.392 × 10^7^	1.276 × 10^8^	9.112 × 10^9^	2.031 × 10^10^	1.113 × 10^8^	8.701 × 10^9^
NPs surface area at well bottom (cm^2^/cm^3^)	0.005	2.656	4.482	2.967	5.550	2.024	4.223
	0.01	5.313	8.964	5.934	11.101	4.048	8.446
NPs surface area per unit area of well (cm^2^/cm^2^)	0.005	0.003	0.004	0.003	0.006	0.002	0.004
	0.01	0.005	0.009	0.006	0.011	0.004	0.008

## Data Availability

All relevant data are included within the paper and its Supporting Information files.

## Supplementary Materials

The following supporting information can be downloaded at: https://www.mdpi.com/article/10.3390/nano12223935/s1, Figure S1: Schema of the NPs suspension as utilized in the DG model. The NPs can be present as individual particles or agglomerates of different sizes (particle numbers and shapes), which have different settling and diffusion rates. The basic geometrical characteristics are shown along with the compartments i − 1, i and i + 1 which reflect differences in NPs concentration within the dispersion as a result of diffusion and sedimentation processes) and the inflow (Min) and outflow (Mout) of solute species (in this case the NPs (free or as agglomerates) between compartments i − 1, i and i + 1. Δz is the distance between the centers of two adjacent compartments. Figure S2: % of number-weighted distributions of hydrodynamic diameters (dH) for AOT-AgNP, PLL-AgNP, CYS-AgNP, GSH-AgNP, CYS-AuNP and GSH-AuNP. Values were taken from Table S2; Table S1: % Number distributions of hydrodynamic diameters (dH), Table S2: Median values of calculated dosimetric parameters for AOT- and PLL- coated silver nanoparticles (AgNP), reported for bottom compartment (cellular microenvironment), for each of the nominal concentrations used in MTT experiments; Table S3: Median values of calculated dosimetric parameters for CYS- and GSH-coated silver and gold nanoparticles (NP), reported for bottom compartment (cellular microenvironment), for each of the nominal concentrations used in MTT experiments.
