# Supplementary material for: An In Vitro Dosimetry Tool for the Numerical Transport Modeling of Engineered Nanomaterials Powered by the Enalos RiskGONE Cloud Platform
Source: Nanomaterials (Basel). 2022 Nov 8;12(22):3935. doi: 10.3390/nano12223935 (PMC9697428; doi:10.3390/nano12223935)
Supplement: Supplementary file 1 [file nanomaterials-12-03935-s001.zip › nanomaterials-1957809-supplementary.pdf]

Supporting Information

1. The Distorted Grid (DG) model

The Distorted Grid model (DG) was presented in detail by DeLoid et al. (18). The model is based on the diffusion and sedimentation of the NPs in well mixed solutions, as shown schematically in Figure S1.

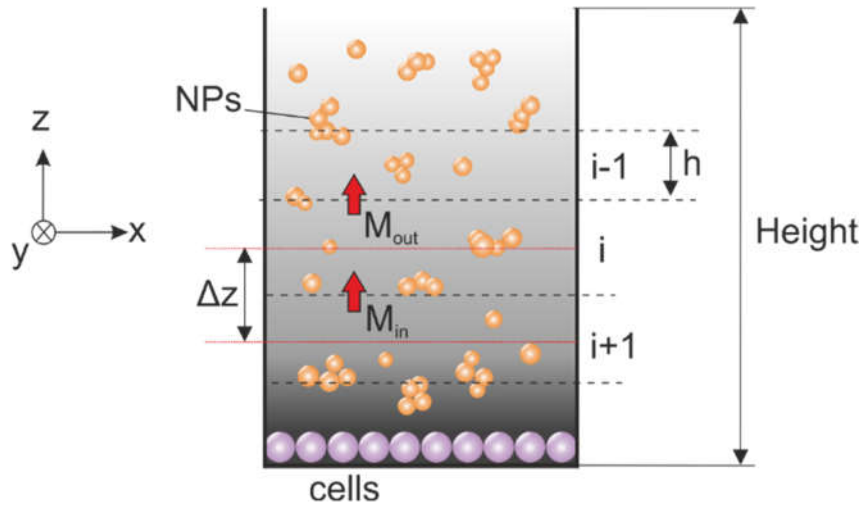

Figure S1. Schema of the NPs suspension as utilized in the DG model. The NPs can be present as individual particles or agglomerates of different sizes (particle numbers and shapes), which have different settling and diffusion rates. The basic geometrical characteristics are shown along with the compartments *i-1, i* and *i+1* which reflect differences in NPs concentration within the dispersion as a result of diffusion and sedimentation processes) and the inflow (M_{in}) and outflow (M_{out}) of solute species (in this case the NPs (free or as agglomerates) between compartments *i-1, i* and *i+1.* Δz is the distance between the centers of two adjacent compartments.

The diffusion of the NPs (and any agglomerates) is assumed to be Fickian i.e., the driving force for the mass transport between the different compartments i, is the concentration gradient,

$$J = -D\frac{\partial C}{\partial z} \tag{1}$$

where C is the concentration of NPs (kg m^{-3}), z is the position (m), and D is the diffusion coefficient (m^2 s^{-1}), which is defined by the Stokes-Einstein equations as,

$$D = \frac{k_B T}{3\pi\eta d_{\mathrm{H}}} \qquad (2)$$

where d_{H} (m) is the particles hydrodynamic diameter, k_B is the Boltzmann constant (kg m^2 s^{-2} K^{-1}), T is the absolute temperature (°K), and η is the media dynamic viscosity (kg m^{-1} s^{-1}). Thus, smaller particles will have a faster diffusion rate than larger ones, or agglomerates.

During a small time interval Δt, the inflow of solute species j (in this case the NP) into compartment i, $M_{in,j,}$ is computed as (see Figure 1):

$$M_{in,i,j} = A\Delta t D_{i+1,j} \frac{C_{i+1,j} - C_{i,j}}{\Delta z} \qquad (3)$$

where A is the cross-sectional area of the column, $D_{i+1,j}$ is the diffusion coefficient of the NP species j at boundary, $C_{i+1,j}$ and $C_{i,j}$ are the concentrations of NP species j in the two compartments, and Δz (see Figure 1) is the distance between the centers of the two compartments. Similarly, the outflow of NPs given as:

$$M_{out,i,j} = A\Delta t D_{i,j} \frac{C_{i-1,j} - C_{i,j}}{\Delta z} \qquad (4)$$

The net change in concentration of species j (the NP) in compartment i, $\Delta C_{i,j}$ is calculated as:

$$\Delta C_{i,j} = \frac{M_{in,i.j} - M_{out,i.j}}{\Delta z} \qquad (5)$$

which from Eqs. (3) and (4) yields,

$$\Delta C_{i,j} = \left(\frac{\Delta t}{\Delta z^2}\right)\left[D_{i,j}\left(C_{i-1,j} - C_{i,j}\right) + D_{i+1,j}\left(C_{i+1,j} - C_{i,j}\right)\right] \tag{6}$$

and the new concentration of species *j* in compartment *i* after time Δt is calculated as:

$$C_{i,j}^{new} = C_{i,j} + \Delta C_{i,j} \tag{7}$$

The sedimentation velocity, v_s, of a NP or agglomerate under gravity is computed as:

$$v_s = \frac{g(\rho_{\mathrm{EV}} - \rho_{\mathrm{media}})d_H^2}{18\eta} \tag{8}$$

where g (m s^{-2}) is the acceleration due to gravity, ρ_{EV} (kg m^{-3}) is the effective density of the particle, and ρ_{media} is the medium density (kg m^{-3}). The sedimentation coefficient of a particle, *S*, is defined as the ratio of a particle's sedimentation velocity to the acceleration applied to it. For a particle under gravity sedimentation is this calculated as:

$$S = \frac{v_s}{g} \tag{9}$$

The downward vertical displacement of a particle with sedimentation coefficient *S* in Δt seconds is determined as:

$$dz = Sg\Delta t \tag{10}$$

In general, sedimentation is modeled by moving the internal boundaries in the direction of sedimentation. However, this approach is not practical, especially for the bottom of the well (i.e., at the bottom of the culture dish) where a number of boundaries would accumulate over the course of the simulation at the

bottom of the well and collapse the associated compartments. In the DG model the NPs concentration in the compartment at the bottom of the well (i.e., that can come into contact with the cells) is of most interest. The sedimentation component of the model, which allows particles to move between the compartments without permanently moving compartment boundaries, is accomplished by calculating, in each round of the simulated sedimentation, the distance by which each compartment i would be displaced during the simulation time interval Δt, for each particle species j:

$$dz_{i,j} = S_{i,j} g \Delta t \tag{11}$$

where $dz_{i,j}$ is the downward displacement of compartment i, and $S_{i,j}$ is the sedimentation coefficient of particle species j at the center (along the z axis) of compartment i. The concentration in compartment i is increased by the product of the concentration in compartment $i - 1$ and the fraction of the compartment height that would displace into compartment i,

$$\Delta C_{in,i,j} = C_{i-1,j} \frac{dz_{i,j}}{h} \tag{12}$$

where h is the height of a compartment. Similarly, by sedimentation of particle species j out of compartment i, the concentration in compartment i is decreased by:

$$\Delta C_{out,i,j} = C_{i,j} \frac{dz_{i,j}}{h} \tag{13}$$

The new concentration is thus calculated for compartment i by adding $\Delta C_{in,i,j}$ to, and subtracting $\Delta C_{out,i,j}$ from its previous concentration, as shown:

$$C_{i,j}^{new} = C_{i-1,j} \frac{dz_{i,j}}{h} + C_{i,j} \frac{dz_{i,j}}{h} \tag{14}$$

The simulation proceeds by alternating rounds of diffusion and sedimentation, each of duration Δt, until the selected cumulative time is obtained. At the end of the simulation, the concentrations in each compartment, as well as the derived mass, particle number and surface area dose metrics are available to the user.

2. Cell cultures

Murine fibroblast cell line L929 (ATCC® CCL- 1TM) was cultured in EMEM (Sigma Aldrich, USA) supplemented with 10% (v/v) FBS (Sigma Aldrich, USA) and 1% (v/v) Pen Strep (Sigma Aldrich, USA) in a T25 culture flask (Eppendorf, Hamburg, Germany). When the cells reached 80% confluence, the culture medium was removed and cells were washed once with sterile phosphate buffer saline (PBS). Cells were then detached from the flask by adding trypsin-EDTA (0.25%) solution (Sigma Aldrich, USA) and incubated for 10 min at 37 °C and 5% CO_2. The detached cells were collected, counted on a TC20 automated cell counter (Biorad, USA), and seeded in sterile 96-well plates (Eppendorf, Germany) in 100 □L/well of cell culture medium. Cells were incubated for 24 h at 37°C and 5% CO_2 to allow cell attachment. The following day, AgNPs and AuNPs suspended in ultra-pure water were added to wells in 1, 5, 10, 50, 100 and 300 mg/L concentrations and incubated for 24 h at 37°C and 5% CO_2. Vehicle control (ultra-pure water) was added to negative control wells, and dimethyl sulfoxide (DMSO) was added to positive control wells to a final concentration of 10% (v/v).

The HaCaT cells were cultured in high glucose Dulbecco's modified Eagle's medium (DMEM) (Sigma Aldrich, St Louis, MO, USA) with addition of 10% heat-inactivated fetal bovine serum (Sigma Aldrich, USA) and 1% penicillin/streptomycin (Sigma Aldrich, USA) in a T75 culture flask (Eppendorf, Hamburg, Germany) until they reached 80% confluency. The culture medium was then removed with a pipette, and cells were washed once with sterile PBS and detached by the addition of 0.25% trypsin–EDTA solution followed by 10 min incubation at 37 °C and 5% CO2. Detached cells were collected, counted on a TC20 automated cell counter (Bio-Rad, Hercules, CA, USA), and seeded in black sterile 96-well plates (Thermo Fisher Scientific, Waltham, MA, USA) at 20,000 cells/well for MTT and dichloro-dihydrofluorescein diacetate (DCFH-DA), assays. Seeded plates were incubated for 24 h at 37 °C and 5% CO2 to allow cell attachment. The following day, NPs were added to wells in different concentrations, while the same volume of sterile Milli-Q water was added to the control cells.

MTT

The MTT assay, based on the reduction of the yellow tetrazolium salt MTT (3-(4,5-dimethylthiazol-2-yl)-2,5-diphenyltetrazolium bromide) to a purple MTT-formazan crystal by metabolically active cells, was used to determine L929 and HaCaT cell viability after 24 h exposure. MTT

reagent (Sigma Aldrich, USA) was dissolved in ultra-pure water to 5 mg/mL stock concentration, which was further diluted to 1 mg/mL working concentration. After 24 h treatment of L929 and HaCaT cells, medium containing NP was aspirated from wells. Cells were then washed three times with PBS (200 µL/well), after which 100 µL/well of fresh cell culture medium was added to plate. MTT solution was added to wells (50 µL/well) and the plate was incubate for 4 h at 37 °C, after which the MTT solution was removed by aspiration. The remaining formazan crystals were dissolved by addition of DMSO (50 µL/well) and shaking the plates. The absorbance was measured at 530 nm using a VictorTM multilabel reader (Perkin Elmer, Massachusetts, USA).

Table S1. % Number distributions of hydrodynamic diameters (d_H)

**% number-weighted size (-)**	**d_H (nm)**					
	**CYS-AuNP**	**GSH-AgNP**	**CYS-AgNP**	**GSH-AuNP**	**AOT-AgNP**	**PLL-AgNP**
**0.4**	0	0	0	0	0	0
**0.463**	0	0	0	0	0	0
**0.536**	0	0	0	0	0	0
**0.621**	0	0	0	0	0	0
**0.719**	0	0	0	0	0	0
**0.833**	0	0	0	0	0	0
**0.965**	0	0	0	0	0	0
**1.12**	0	0	0	0	0	0
**1.29**	0	0	0	0	0	0
**1.5**	0	0	0	0	0	0
**1.74**	0	0	0	0	0	0
**2.01**	0	0	0	0	0	0
**2.33**	0	0	0	0	0	0
**2.7**	0	0	0	0	0	0
**3.12**	0	7.18	0	6.96	0	0
**3.62**	0	22.6	0	22.8	0	0
**4.19**	0	29	7.38	30.1	0	0
**4.85**	0	21.5	22.9	22	0	0
**5.61**	0	11.6	29	10.8	0	0
**6.5**	0	5.06	21.3	4.2	0	0
**7.53**	0	1.92	11.5	1.57	0	0
**8.72**	0	0.684	5.06	0.719	0	0
**10.1**	0	0.251	1.92	0.413	0	0
**11.7**	1.74	0.101	0.648	0.241	0	0
**13.5**	10	0.043	0.201	0.126	0	0
**15.7**	21.4	0.0173	0.0588	0.0556	0	4.94

**18.2**	24.1	0.006	0.0165	0.0205	0	17.2
**21**	18.1	0.00159	0.00434	0.00601	0	25.7
**24.4**	11.1	0.00026	0.00097	0.00128	0	23
**28.2**	6.23	1.8E-05	0.00015	0.00015	2.71	15.1
**32.7**	3.41	0	1.12E-05	2.07E-05	11.5	8.03
**37.8**	1.86	0	0	7.57E-05	21.1	3.64
**43.8**	1.02	0	0	0.00012	23.1	1.45
**50.7**	0.549	0	0	0.00013	18.2	0.522
**58.8**	0.29	0	0	9.93E-05	11.6	0.177
**68.1**	0.149	0	5.5E-08	6.64E-05	6.36	0.0646
**78.8**	0.0744	0	3.1E-07	3.96E-05	3.13	0.0304
**91.3**	0.0364	0	7.2E-07	2.19E-05	1.41	0.0192
**106**	0.0176	0	9.2E-07	1.15E-05	0.587	0.014
**122**	0.0086	0	8.9E-07	5.88E-06	0.226	0.0106
**142**	0.0044	0	7.8E-07	3.06E-06	0.0802	0.00849
**164**	0.00236	4.7E-09	6.6E-07	1.62E-06	0.0247	0.00696
**190**	0.0012	2.7E-08	5.1E-07	7.79E-07	0.00543	0.0053
**220**	0.00051	6.2E-08	3.6E-07	3.02E-07	0.000496	0.00363
**255**	0.00019	8.3E-08	2.5E-07	9.31E-08	0	0.00237
**295**	6.1E-05	7.5E-08	1.9E-07	2.00E-08	0	0.00153
**342**	1.4E-05	4.9E-08	1.4E-07	2.19E-09	0	0.00098
**396**	1.8E-06	2.2E-08	1.1E-07	0	0	0.00060
**459**	0	5.5E-09	7.9E-08	0	0	0.00034
**531**	0	3.9E-10	5.4E-08	0	0	0.00017
**615**	0	0	3.4E-08	0	0	0.00007
**712**	0	0	1.8E-08	0	0	0.00002
**825**	0	0	7.5E-09	0	0	4.1E-06
**955**	0	0	1.8E-09	0	0	0
**1.11E+03**	0	0	1.4E-10	0	0	0
**1.28E+03**	0	0	0	0	0	0
**1.48E+03**	0	0	0	0	0	0
**1.72E+03**	0	0	0	0	0	0
**1.99E+03**	0	0	0	0	0	0
**2.30E+03**	0	0	0	0	0	0
**2.67E+03**	0	0	0	0	0	0
**3.09E+03**	0	0	0	0	0	0
**3.58E+03**	0	0	0	0	0	0
**4.15E+03**	0	0	1.1E-11	0	0	0
**4.80E+03**	0	0	5.4E-11	0	0	0
**5.56E+03**	0	0	7.6E-11	0	0	0
**6.44E+03**	0	0	3.2E-11	0	0	0
**7.46E+03**	0	0	0	0	0	0
**8.63E+03**	0	0	0	0	0	0

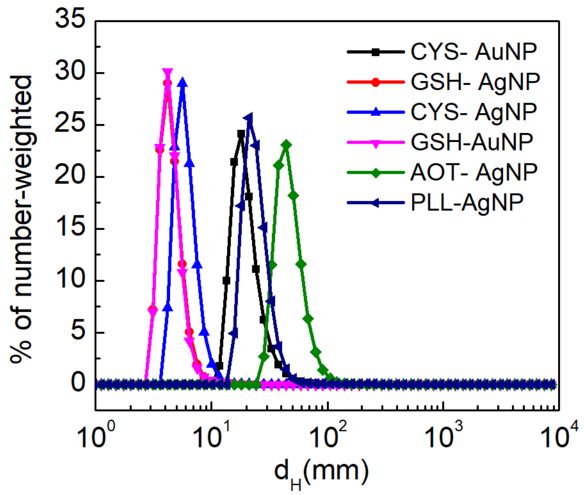

Figure S2 % of number-weighted distributions of hydrodynamic diameters (d_H) for AOT-AgNP, PLL-AgNP, CYS-AgNP, GSH-AgNP, CYS-AuNP and GSH-AuNP. Values were taken from Table S2.

Table S2. Median values of calculated dosimetric parameters for AOT- and PLL- coated silver nanoparticles (AgNP), reported for bottom compartment (cellular microenvironment), for each of the nominal concentrations used in MTT experiments.

Calculated value	Nanoparticle	Nominal concentration (mg/cm^3)							
		**0.16**	**0.08**	**0.04**	**0.02**	**0.01**	**0.005**	**0.0025**	**0.00125**
**mass of AgNP at bottom compartment (mg/cm^3)**	AOT AgNP	1.341	0.671	0.335	0.168	0.084	0.042	0.021	0.010
	PLL AgNP	6.051	3.026	1.513	0.756	0.0378	0.189	0.095	0.047
**mass per unit area of well (mg/cm^2)**	AOT AgNP	$1.341*10^{-3}$	$6.707*10^{-4}$	$3.354*10^{-4}$	$1.677*10^{-4}$	$8.384*10^{-5}$	$4.192*10^{-5}$	$2.096*10^{-5}$	$1.048*10^{-5}$
	PLL AgNP	$6.051*10^{-3}$	$3.026*10^{-3}$	$1.513*10^{-3}$	$7.564*10^{-4}$	$3.782*10^{-4}$	$1.891*10^{-4}$	$9.455*10^{-5}$	$4.727*10^{-5}$
**AgNP number at bottom compartment (cm^{-3})**	AOT AgNP	$7.028*10^{11}$	$3.514*10^{11}$	$1.757*10^{11}$	$8.785*10^{10}$	$4.392*10^{10}$	$2.196*10^{10}$	$1.098*10^{10}$	$5.490*10^{9}$
	PLL AgNP	$2.042*10^{12}$	$1.021*10^{12}$	$5.106*10^{11}$	$2.553*10^{11}$	$1.276*10^{11}$	$6.382*10$	$3.191*10^{10}$	$1.595*10^{10}$
**AgNP number per unit area of well bottom (cm^{-2})**	AOT AgNP	$7.028*10^{8}$	$3.514*10^{8}$	$1.757*10^{8}$	$8.785*10^{7}$	$4.392*10^{7}$	$2.196*10^{7}$	$1.098*10^{7}$	$5.490*10^{6}$
	PLL AgNP	$2.042*10^{9}$	$1.021*10^{9}$	$5.106*10^{8}$	$2.553*10^{8}$	$1.276*10^{8}$	$6.382*10^{7}$	$3.191*10^{7}$	$1.595*10^{7}$
**AgNP surface area at bottom compartment (cm^2/cm^3)**	AOT AgNP	85.001	42.501	21.250	10.625	5.313	2.656	1.328	0.664
	PLL AgNP	143.426	71.713	35.857	17.928	8.964	4.482	2.241	1.121
**AgNP surface area per unit area of well (cm^2/cm^2)**	AOT AgNP	0.085	0.043	0.021	0.011	0.005	0.003	0.001	$7*10^{-4}$
	PLL AgNP	0.143	0.072	0.036	0.018	0.009	0.004	0.002	0.001

Table S3. Median values of calculated dosimetric parameters for CYS- and GSH-coated silver and gold nanoparticles (NP), reported for bottom compartment (cellular microenvironment), for each of the nominal concentrations used in MTT experiments.

Calculated value	NP type	Nominal concentration (mg/cm^3)					
		**0.3**	**0.1**	**0.05**	**0.01**	**0.005**	**0.001**
**mass of NP at bottom compartment (mg/cm^3)**	GSH-AuNP	0.501	0.167	0.084	0.017	0.008	0.002
	GSH-AgNP	0.301	0.100	0.050	0.010	0.005	0.001
	CYS AuNP	3.406	1.135	0.568	0.114	0.057	0.011
	CYS AgNP	0.302	0.101	0.050	0.010	0.005	0.001
	GSH-AuNP	$5.012*10^{-4}$	$1.671*10^{-4}$	$8.353*10^{-5}$	$1.671*10^{-5}$	$8.353*10^{-6}$	$1.671*10^{-6}$

**mass per unit area of well (mg/cm^2)**	GSH-AgNP	$3.011*10^{-4}$	$1.004*10^{-4}$	$5.019*10^{-5}$	$1.004*10^{-5}$	$5.019*10^{-6}$	$1.004*10^{-6}$
	CYS AuNP	$3.406*10^{-3}$	$1.135*10^{-3}$	$5.676*10^{-4}$	$1.135*10^{-4}$	$5.676*10^{-5}$	$1.135*10^{-5}$
	CYS AgNP	$3.021*10^{-4}$	$1.007*10^{-4}$	$5.034*10^{-5}$	$1.007*10^{-5}$	$5.034*10^{-6}$	$1.007*10^{-6}$
**NP number at bottom compartment (cm^{-3})**	GSH-AuNP	$2.734*10^{14}$	$9.112*10^{13}$	$4.556*10^{13}$	$9.112*10^{12}$	$4.556*10^{12}$	$9.112*10^{11}$
	GSH-AgNP	$6.093*10^{14}$	$2.031*10^{14}$	$1.016*10^{14}$	$2.031*10^{13}$	$1.016*10^{13}$	$2.031*10^{12}$
	CYS AuNP	$3.339*10^{12}$	$1.113*10^{12}$	$5.564*10^{11}$	$1.113*10^{11}$	$5.564*10^{10}$	$1.113*10^{10}$
	CYS AgNP	$2.610*10^{14}$	$8.701*10^{13}$	$4.350*10^{13}$	$8.701*10^{12}$	$4.350*10^{12}$	$8.701*10^{11}$
**NP number per unit area of well bottom (cm^{-2})**	GSH-AuNP	$2.734*10^{11}$	$9.112*10^{10}$	$4.556*10^{10}$	$9.112*10^{9}$	$4.556*10^{9}$	$9.112*10^{8}$
	GSH-AgNP	$6.093*10^{11}$	$2.031*10^{11}$	$1.016*10^{11}$	$2.031*10^{10}$	$1.016*10^{10}$	$2.031*10^{9}$
	CYS AuNP	$3.339*10^{9}$	$1.113*10^{9}$	$5.564*10^{8}$	$1.113*10^{8}$	$5.564*10^{7}$	$1.113*10^{7}$
	CYS AgNP	$2.610*10^{11}$	$8.701*10^{10}$	$4.350*10^{10}$	$8.701*10^{9}$	$4.350*10^{9}$	$8.701*10^{8}$
**NP surface area at bottom compartment (cm^2/cm^3)**	GSH-AuNP	178.031	59.344	29.672	5.934	2.967	0.593
	GSH-AgNP	333.018	111.006	55.503	11.101	5.550	1.110
	CYS AuNP	121.436	40.479	20.239	4.048	2.024	0.405
	CYS AgNP	253.388	84.463	42.231	8.446	4.223	0.845
**NP surface area per unit area of well (cm^2/cm^2)**	GSH-AuNP	0.178	0.059	0.030	0.006	0.003	0.001
	GSH-AgNP	0.333	0.111	0.056	0.011	0.006	0.001
	CYS AuNP	0.121	0.040	0.020	0.004	0.002	$4*10^{-4}$
	CYS AgNP	0.253	0.084	0.042	0.008	0.004	0.001
